# Lifestyle patterns and incident type 2 diabetes in the Dutch lifelines cohort study

**DOI:** 10.1016/j.pmedr.2022.102012

**Published:** 2022-10-03

**Authors:** Ming-Jie Duan, Louise H. Dekker, Juan-Jesus Carrero, Gerjan Navis

**Affiliations:** a Department of Internal Medicine, University Medical Center Groningen, Groningen, The Netherlands; b National Institute for Public Health and the Environment, Bilthoven, The Netherlands; c Department of Medical Epidemiology and Biostatistics, Karolinska Institutet, Stockholm, Sweden

**Keywords:** Lifestyle patterns, Lifestyle, Type 2 diabetes, Epidemiology, Public health, BIC-LL, Bayesian information criterion with log likelihood for the number of parameters adjusted, FFQ, Food frequency questionnaire, LCA, Latent class analysis, LLDS, Lifeline diet score, MVPA, Moderate-to-vigorous physical activity, PAF, Population attributable fraction, SQUASH, Short QUestionnaire to ASsess Health-enhancing physical activity

## Abstract

We aimed to identify the underlying subgroups of the population characterized by distinct lifestyle patterns, and to investigate the associations between lifestyle patterns and risk of incident type 2 diabetes. Using data from the Dutch Lifelines cohort study, latent class analysis was performed to derive lifestyle patterns on five lifestyle factors, i.e., smoking, diet quality, TV watching time, physical activity level, and risk drinking. Associations between lifestyle patterns and incident type 2 diabetes were estimated. Among 61,869 participants analyzed, we identified 900 cases of type 2 diabetes during follow-up (205,696 person-years; incidence rate 4.38 per 1000 person-years). Five lifestyle pattern groups were identified. Using the “healthy lifestyle group” as reference, the “unhealthy lifestyle group” had the highest risk for type 2 diabetes (HR 1.51 [95%CI 1.24, 1.85]), followed by the “poor diet and low physical activity group” (HR 1.26 [95%CI 1.03, 1.55]). The “risk drinker group” and the “couch potato group” (characterized by excessive TV watching) showed no significantly elevated risk. These models were adjusted for age, sex, total energy intake, education, BMI, family history of diabetes, and blood glucose level at baseline. Our study shows that lifestyle factors tended to cluster in unique behavioral patterns within the heterogeneous population. These lifestyle patterns were differentially associated with incident type 2 diabetes. Our findings support the relevance of considering lifestyle patterns in type 2 diabetes prevention. Tailored prevention strategies that target multiple lifestyle risk factors for different lifestyle pattern groups may optimize the effectiveness of diabetes prevention at the population level.

## Highlights

• Lifestyle factors clustered in behavioral patterns within the population.
• Different lifestyle patterns were differentially associated with risk of developing type 2 diabetes.
• A lifestyle pattern may be a proxy for an underlying variable that is relevant for the prevention of type 2 diabetes.

## 1 Introduction

Type 2 diabetes is a major public health challenge that leads to considerable morbidity, mortality, and economic burden (Sun et al., 2022). Lifestyle is crucial to the prevention of type 2 diabetes. Adherence to a combination of healthy lifestyle factors – healthy diet, avoiding smoking, vigorous physical activity – is found to substantially lower the risk of developing type 2 diabetes (Duan et al., 2022, Farhadnejad et al., 2022, Zhang et al., 2020).

For studying the relationships between lifestyle factors and type 2 diabetes, a single lifestyle factor approach has been widely applied. Studies have also examined the combined effects of lifestyle factors, such as using an unweighted lifestyle score, but they do not take account of the distribution of the lifestyle factors in the population (Zhang et al., 2020). Prior studies have implicated that lifestyle factors often co-occur in behavioral patterns and may have interdependent effects on health (Davis et al., 2019, Ding et al., 2015, Hendryx et al., 2020, Hofstetter et al., 2014, Luo et al., 2021, Meader et al., 2016, Morris et al., 2016, Noble et al., 2015, Poortinga, 2007, van Etten et al., 2020, Watts et al., 2016). Better methodological approaches are therefore needed to understand the complexities of lifestyle factors and their associations with health.

For type 2 diabetes prevention, current evidence supports the relevance of targeting multiple lifestyle risk factors simultaneously (Meader et al., 2016, Noble et al., 2015, Tuomilehto et al., 2011). It is therefore essential to have a clear understanding of the clustering of lifestyle risk factors of the target populations. However, to date the knowledge basis is lacking. Specifically, only three studies have identified lifestyle patterns in the Dutch population, and only one of them further studied their associations with risk of type 2 diabetes (de Vries et al., 2008, Hofstetter et al., 2014, van Etten et al., 2020). There is considerably less knowledge about the relevance of lifestyle patterns for type 2 diabetes prevention in the general population.

Previous studies on lifestyle patterns mainly included smoking, alcohol consumption, physical activity level, and fruit and vegetable intake (Davis et al., 2019, de Vries et al., 2008, Hendryx et al., 2020, Hofstetter et al., 2014, Luo et al., 2021, Poortinga, 2007, van Etten et al., 2020). However, those identified lifestyle patterns may not fully represent the overall lifestyle risk profiles. While fruit and vegetable intake is an important indicator of diet (Halvorsen et al., 2021), overall diet quality, commonly assessed by diet scores, may better represent the overall dietary “risk profile” of the target populations (Vinke et al., 2018). Moreover, high TV watching time, as an emerging lifestyle risk factor representing sedentary behavior, has been found to be a risk factor for type 2 diabetes and mortality, independent of moderate-to-vigorous physical activity (MVPA) (Patterson et al., 2018), while it has never been included in lifestyle pattern analysis. Therefore, incorporating overall diet quality and TV watching time in lifestyle pattern analysis will provide more information on the clinical relevance of lifestyle patterns.

Using a large Dutch population cohort, we aimed to reveal how lifestyle factors cluster within populations, i.e., the diverse lifestyle risk patterns of the population, and subsequently, to investigate the prospective associations between lifestyle patterns and incident type 2 diabetes. The analysis focused on four traditional and one emerging lifestyle factors, including overall diet quality (Duan et al., 2021, Maghsoudi et al., 2016, Vinke et al., 2018), physical activity (Aune et al., 2015), smoking (Pan et al., 2015), risk drinking (Knott et al., 2015), and TV watching time (Patterson et al., 2018). These lifestyle factors included are common in the general population. Having a clear understanding of how these common lifestyle factors cluster and how different lifestyle clusters affect type 2 diabetes risk will facilitate the design of effective prevention strategies at population level.

## 2 Methods

### 2.1 Study design and population

The Lifelines cohort study is a multidisciplinary prospective population-based cohort study that applies a unique three-generation design to study the health and health-related behaviors of 167,729 persons living in the north of The Netherlands. Before study entry, a signed informed consent form was obtained from each participant. The Lifelines study is conducted according to the principles of the Declaration of Helsinki and approved by the Medical Ethics Committee of the University Medical Center Groningen, The Netherlands. The overall design and rationale of the study have been described in detail elsewhere (Klijs et al., 2015, Scholtens et al., 2015).

Participants were included in the study between 2006 and 2013. So far, four assessment rounds took place, including baseline assessment (T1) and three follow-ups (T2-T4). Comprehensive physical examinations, biobanking, and questionnaires were conducted at T1 and T4. Follow-up questionnaires were issued to participants at T2, T3, and T4.

Participants aged between 35 and 65 years who were free of diabetes at baseline, and for whom lifestyle data was available were included in this study. Participants who had no follow-up data, or who reported the development of type 1 diabetes or gestational diabetes during follow-up were excluded. In total, 61,869 participants were included in the analysis (Supplementary Fig. S1).

### 2.2 Ascertainment of incident type 2 diabetes

Incident type 2 diabetes was assessed by self-report questionnaires during follow-up at T2, T3, and T4, as well as blood glucose and HbA_1c_ measurements at T4. Blood measurements are not available at T2 and T3. Participants were considered an incident case if they met one of the following criteria: (1) self-reported newly developed type 2 diabetes since last time they filled out a questionnaire; (2) fasting blood glucose ≥ 7.0 mmol/L; or (3) HbA_1c_ ≥ 48 mmol/mol (6.5 %) (World Health Organization (WHO) and International Diabetes Federation (IDF), 2006).

### 2.3 Clinical measurements

Blood samples were collected by venipuncture in a fasting state, and were further transferred to the Lifelines central laboratory for analysis. Serum levels of glucose and HbA_1c_ were subsequently analyzed. Anthropometry was measured by trained research staff following standardized protocols. These measurements were performed without shoes and heavy clothing. Family history of diabetes was assessed by self-administered questionnaires. Participants were considered having a family history of diabetes if they reported having a first-degree relative (i.e., parent, sibling, or child) ever being diagnosed with type 2 diabetes.

### 2.4 Assessment of lifestyle factors and sociodemographic covariates

Age, smoking status, TV watching time per day, and education were assessed by self-administered questionnaires. Highest education achieved was categorized as: (1) low – junior general secondary education or lower; (2) middle – secondary vocational education and senior general secondary education; and (3) high – higher vocational education or university.

Habitual physical activity level of a normal week was assessed by the Short QUestionnaire to ASsess Health-enhancing physical activity (SQUASH). The SQUASH was pre-structured into four domains: commuting, leisure time, household, and occupational activities. For each reported activity, frequency (days per week) and duration (average time per day) were asked. From the SQUASH data, non-occupational moderate-to-vigorous physical activity (MVPA), including commuting and sports (if ≥ 4.0 MET), was calculated in minutes per week. The SQUASH has been validated in the general population using objective accelerometer measurements for a 2-week period (Wendel-Vos et al., 2003).

Dietary intake was assessed by a semi-quantitative self-administered food frequency questionnaire (FFQ). The FFQ aimed to assess the habitual intake of 110 food items (including alcohol) during the past 4 weeks. For 46 main food items (such as bread and milk), frequency of consumption was indicated as ‘not this month’ or in days per week or month, including the amount (in units or specified portion sizes) consumed each time. The FFQ also included 37 questions on intake of sub-items (such as different types of cheese) for which frequency was specified as never, sometimes, often, and always. The FFQ was designed based on the validated Dutch FFQ (Molag et al., 2010). In brief, the intake of the food items and the energy intake have been tested and validated against three 24-h dietary recalls and actual energy intake in controlled feeding trials, respectively (Siebelink et al., 2011, Streppel et al., 2013). The Lifelines Diet Score (LLDS) was calculated to evaluate the relative diet quality of each participant (Vinke et al., 2018).

### 2.5 Statistical analysis

#### 2.5.1 Lifestyle pattern analysis with latent class analysis

Lifestyle patterns were derived using latent class analysis (LCA). LCA is a latent variable mixture model that relates a set of observed indicators (i.e., lifestyle variables) to a set of latent variables (i.e., lifestyle pattern classes) (Hagenaars and McCutcheon, 2002). LCA enables the analysis and interpretation of higher-order interactions among lifestyle factors, which overcomes the issue of collinearity between lifestyle factors (Lanza and Rhoades, 2013, Rabe-Hesketh and Skrondal, 2008).

The LCA output mainly consists of two parts. The first part is the posterior class probability, which estimates the probability of an individual belonging to each latent class given the individual’s observed response on the measured indicators. Each participant was assigned to the lifestyle pattern group for which they had the highest posterior class probability. A number of mutually exclusive lifestyle pattern groups would thus be identified. The second part is the class-specific response probability, which estimates the likelihood that an individual, who belongs to a particular latent class, adheres to a certain measured indicator, such as the probability of being a never smoker (Hagenaars and McCutcheon, 2002).

Since LCA requires that items are measured categorically, we further defined lifestyle factors into risky versus non-risky categories based on evidence, resulting in nine indicators. The interpretation of the results also becomes clearer when lifestyle factors are categorized into risky versus non-risky groups. Specifically, smoking status, i.e., never, former, and current smoker, was treated as three dummy variables. Alcohol intake was categorized as risk drinking (>15 g alcohol/day) versus non-risk drinking (≤15 g alcohol/day) (Ding et al., 2021). This amount was approximated to one drink per day. TV watching time was categorized as excessive TV watching (highest sex-specific tertile) versus non-excessive TV watching (other tertiles). LLDS was divided into sex-specific tertiles. Physical activity level was categorized as whether the participant met the Dutch recommendation for physical activity level, i.e., ≥150 min non-occupational MVPA per week (Weggemans et al., 2018).

A series of latent class models were examined with three through nine classes. We selected the best-fitting latent class solution based on Bayesian information criterion with log likelihood for the number of parameters adjusted (BIC-LL). BIC-LL is a model goodness-of-fit index, for which a lower value is preferred (Nylund et al., 2007). We also considered other model goodness-of-fit indices (**Supplementary** Table S1) (Hagenaars and McCutcheon, 2002), as well as the interpretability of the identified lifestyle patterns. LCA was performed with LatentGOLD (version 5.0.0.14260; Statistical Innovations Inc., Belmont, MA, USA) (Vermunt and Magidson, 2005).

### 2.6 Risk of type 2 diabetes

Associations between lifestyle patterns and incident type 2 diabetes were estimated using Cox proportional hazards regression models. Non-diabetes cases were censored at the last time-point, for which data was available. Additionally, all participants were censored after 60 months. Analyses were adjusted in a stepwise manner for (1) age, sex, and total energy intake; (2) education; (3) BMI; (4) family history of diabetes; and (5) blood glucose level at baseline. Proportional hazards assumption was assessed by calculating the Schoenfeld residuals and by performing Cox regression models with time-dependent covariates. Potential effect modification was evaluated for age, sex, BMI, education, and family history of diabetes. Analyses were repeated excluding participants who had less than 12-month follow-up, in an attempt to address possible reverse causation caused by short follow-up time. For comparisons, we additionally tested the associations of incident type 2 diabetes with each lifestyle risk factor separately. Statistical analyses for calculating the risk of type 2 diabetes were performed on Stata (version 13.1; StataCorp, College Station, TX, USA).

To obtain insights into the lifestyle-related diabetes disease burden, namely the fraction of cases preventable if having a healthy lifestyle profile, we calculated the adjusted population attributable fraction (PAF) based on the odds ratios estimated using logistic regression models adjusting for the abovementioned Cox proportional hazards model covariates. The calculation of PAFs was performed using *punaf* package in Stata, as described by Newson (Newson, 2013).

## 3 Results

### 3.1 Lifestyle patterns

After examining models with three through nine latent classes, we selected a 5-latent class model (five lifestyle patterns) since it offered the lowest BIC-LL value (best model fit) and the best subjective interpretability. Most of the other model goodness-of-fit indices also showed their best values at the 5-latent class model solution. **Supplementary** Table S1 shows the detailed model goodness-of-fit indices for all models tested.

**Fig. 1:**
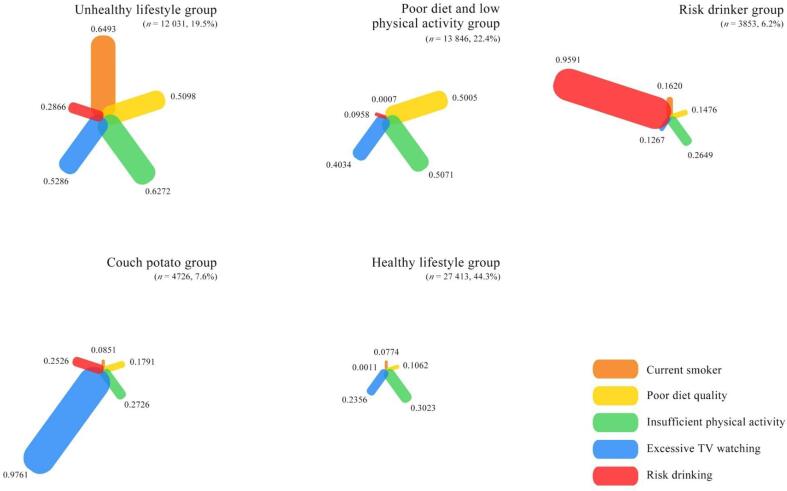
Estimated probabilities of adhering to examined lifestyle risk factors for each identified lifestyle pattern^*, *^The adapted spider charts show the estimated probabilities of adhering to the examined lifestyle risk factors according to each lifestyle pattern, in which the width and the length of each bar was proportionately illustrated according to the values of the estimated probabilities that are displayed next to each bar.

Fig. 1 and **Supplementary** Table S2 show the estimated probabilities of adhering to lifestyle factors for lifestyle patterns identified. The first pattern was named the “healthy lifestyle group” (*n* = 27,413, 44.3 %), as it was characterized by moderate to low probabilities across all lifestyle risk factors. The second pattern was designated as the “poor diet and low physical activity group” (*n* = 13,846, 22.4 %), because it was characterized primarily by moderate to high probabilities of poor diet quality (lowest tertile of LLDS) and insufficient physical activity. The third pattern was labelled the “unhealthy lifestyle group” (*n* = 12,031, 19.5 %), since it was characterized by moderate to low probabilities of risk drinking and former smoker, but moderate to high probabilities across all other lifestyle risk factors. The fourth pattern was named the “couch potato group” (*n* = 4726, 7.6 %). Persons in this pattern had moderate to high probabilities of excessive TV watching and also notably former smoker, but they had moderate to low probabilities elsewhere. The fifth pattern was labelled the “risk drinker group” (*n* = 3853, 6.2 %), as persons in this pattern mainly had very high probability of risk drinking and moderate to high probability of former smoker.

### 3.2 Baseline characteristics

Baseline characteristics for each lifestyle pattern group are shown in Table 1. Participants from the “poor diet and low physical activity group” and the “unhealthy lifestyle group” tended to be younger, while participants from the latter group and the “couch potato group” tended to be less educated. In total, there were 59.6 % female participants included in the analysis, whereas there were more male participants (61.1 %) in the “risk drinker group”. Clinical biomarkers showed diverse distributions among different groups. The “couch potato group” had the highest prevalence of family history of diabetes (10.2 %).

**Table 1:** Baseline characteristics according to lifestyle pattern groups*.

	**Healthy lifestyle group**	**Poor diet and low physical activity group**	**Unhealthy lifestyle group**	**Couch potato group**	**Risk drinker group**	**Total**
Number of participants	27,413	13,846	12,031	4726	3853	61,869
Class size, %	44.3	22.4	19.5	7.6	6.2	100
**Socio-demographic characteristics**						
Age, yrs	48.8 ± 8.0	45.6 ± 7.2	47.5 ± 7.5	51.8 ± 8.1	50.5 ± 7.8	48.2 ± 7.9
Sex - women, %	63.0	61.8	57.1	56.9	38.9	59.6
Education, %						
Low	23.9	26.3	40.9	42.3	19.3	28.8
Middle	38.2	43.8	41.0	37.0	33.6	39.6
High	37.8	29.6	17.9	20.2	46.8	31.3
**Clinical biomarkers**						
Fasting glucose, mmol/L	4.91 ± 0.49	4.93 ± 0.49	5.00 ± 0.52	5.07 ± 0.52	5.05 ± 0.51	4.95 ± 0.50
HbA_1c_, %	5.53 ± 0.29	5.51 ± 0.30	5.58 ± 0.29	5.56 ± 0.30	5.52 ± 0.29	5.54 ± 0.30
HbA_1c_, mmol/mol	36.96 ± 3.22	36.71 ± 3.23	37.50 ± 3.21	37.30 ± 3.30	36.84 ± 3.22	37.03 ± 3.24
BMI, kg/m^2^	25.8 ± 3.9	26.4 ± 4.4	26.5 ± 4.3	26.9 ± 3.9	25.8 ± 3.3	26.1 ± 4.1
Family history of diabetes, %	8.6	8.9	9.3	10.2	7.0	8.8
**Lifestyle factors**						
Total energy intake, kcal/day	1944 ± 540	2158 ± 598	2150 ± 632	2109 ± 589	2218 ± 607	2062 ± 590
Lifeline diet score	27.8 ± 4.8	19.9 ± 3.9	19.9 ± 4.6	22.9 ± 4.9	25.9 ± 4.9	24.0 ± 5.9
Lowest tertile, %	4.5	56.2	57.3	29.8	9.5	28.6
Middle tertile, %	31.4	43.8	36.8	52.1	46.2	37.7
Highest tertile, %	64.2	0	5.9	18.1	44.3	33.7
Risk drinking, %	0	8.5	27.6	39.1	100	16.5
Alcohol intake, g/day	3.3 (0.9, 6.9)	2.4 (0, 6.7)	6.6 (1.5, 16.4)	9.5 (2.6, 17.9)	18.2 (17.3, 25.4)	4.5 (0.9, 11.0)
Meeting physical recommendation (150 min/wk MVPA)	73.9	44.4	22.7	94.4	79.1	59.2
MVPA, min/wk	250 (135, 420)	120 (50, 280)	60 (0, 130)	305 (210, 495)	270 (160, 450)	180 (60, 360)
Excessive TV watching (highest tertile), %	20.9	46.3	61.2	100	0	39.1
TV watching time, hrs/day	2.0 ± 1.1	2.6 ± 1.3	3.0 ± 1.4	3.7 ± 0.9	1.6 ± 0.7	2.4 ± 1.3
Smoking, %						
Never	46.2	100	0	9.6	21.9	44.9
Former	45.4	0	32.8	90.4	64.1	37.4
Current	8.4	0	67.2	0	14.0	17.7

* Data are expressed as unadjusted mean ± standard deviation for age, fasting glucose, HbA_1c_, body mass index (BMI), total energy intake, Lifelines diet score (0–48 no unit), and TV watching time; data are expressed as median (interquartile) for alcohol intake and non-occupational moderate-to-vigorous physical activity level (MVPA); data are expressed as actual observed values for other variables.

### 3.3 Risk of incident type 2 diabetes

Table 2 shows the associations between different lifestyle pattern groups and risks of incident type 2 diabetes. Among 61,869 participants included in the analysis, we identified 900 cases of type 2 diabetes during follow-up (205,696 person-years; median [interquartile] follow-up time, 41 [29–50] months; incidence rate 4.38 per 1000 person-years). The incidence rates of type 2 diabetes ranged from 3.51 per 1000 person-years for the “healthy lifestyle group” to 6.42 per 1000 person-years for the “unhealthy lifestyle group”. In the fully adjusted model (model 5) using the “healthy lifestyle group” as the low risk reference group, the “risk drinker group” (HR 1.03 [95 %CI 0.77, 1.39]) and the “couch potato group” (HR 0.98 [95 %CI 0.76, 1.25]) were not associated with incident type 2 diabetes, whereas the “poor diet and low physical activity group” (HR 1.26 [95 %CI 1.03, 1.55]) and the “unhealthy lifestyle group” (HR 1.51 [95 %CI 1.24, 1.85]) had significantly higher risks of incident type 2 diabetes. **Supplementary** Table S3 shows the associations using the “unhealthy lifestyle group” as reference. Statistically, the associations between lifestyle pattern groups and risks of incident type 2 diabetes were not significantly modified by age, sex, BMI, education, and family history of diabetes (all *p*_interaction_ > 0.05). Results were basically unchanged when excluding participants who had less than 12-month follow-up (**Supplementary** Table S4). **Supplementary** Table S5 presents the PAFs for each lifestyle pattern group using the “healthy lifestyle group” as reference. **Supplementary** Table S6 shows the associations between single lifestyle factors and incident type 2 diabetes.

**Table 2:** Associations between lifestyle pattern groups and incident type 2 diabetes*.

	**Healthy lifestyle group**	**Poor diet and low physical activity group**	**Unhealthy lifestyle group**	**Couch potato group**	**Risk drinker group**
Cases/Population	321 / 27,413	187 / 13,846	255 / 12,031	81 / 4726	56 / 3853
Incidence, %	1.17	1.35	2.12	1.71	1.45
Incidence rates, per 1000 person-years	3.51	4.09	6.42	5.17	4.25
Model 1	1.00 (ref)	1.46 (1.22, 1.75)	1.99 (1.68, 2.34)	1.28 (1.00, 1.64)	0.99 (0.74, 1.32)
Model 2		1.40 (1.17, 1.69)	1.80 (1.51, 2.13)	1.18 (0.92, 1.51)	1.05 (0.79, 1.39)
Model 3		1.22 (1.01, 1.47)	1.64 (1.38, 1.94)	1.06 (0.83, 1.36)	1.09 (0.82, 1.45)
Model 4		1.21 (1.00, 1.46)	1.63 (1.37, 1.93)	1.05 (0.82, 1.35)	1.11 (0.83, 1.47)
Model 5		1.26 (1.03, 1.55)	1.51 (1.24, 1.85)	0.98 (0.76, 1.25)	1.03 (0.77, 1.39)

* All models: HRs (95 % CI) derived from multivariate Cox proportional hazards models. Model 1 was adjusted for age, sex, and total energy intake, *n* = 61,869; model 2 was adjusted for model 1 covariates plus education, *n* = 61,714; model 3 was adjusted for model 2 covariates plus BMI, *n* = 61,714; model 4 was adjusted for model 3 covariates plus family history of diabetes, *n* = 61,714; model 5 was adjusted for model 4 covariates plus blood glucose level at baseline, *n* = 61,512.

## 4 Discussion

There are two main findings of our study. First, using a large population-based sample, we identified five lifestyle patterns. Second, we found that different combinations of lifestyle risk factors, as manifested in lifestyle patterns, were differentially associated with risk of developing type 2 diabetes.

### 4.1 Lifestyle patterns and risk of incident type 2 diabetes

There is robust evidence showing that avoiding risky lifestyle behaviors is effective in the prevention of type 2 diabetes (Farhadnejad et al., 2022, Zhang et al., 2020). For example, an Iranian study found that a higher healthy lifestyle score, characterized by no smoking, normal body weight, vigorous physical activity, and healthy diet, was associated with up to 75 % lower risk of type 2 diabetes, independent of multiple confounders (Farhadnejad et al., 2022). The current analysis extends previous knowledge by considering multiple co-occurring lifestyle risk factors simultaneously in the form of real-life lifestyle patterns in the general population. We are aware of only two other studies that have applied a lifestyle pattern approach when predicting the risk of type 2 diabetes. One study from the US Women’s Health Initiative cohort found that the “poor diet and low exercise pattern” and the “high multiple lifestyle and psychosocial risks pattern” were associated with higher risks of incident type 2 diabetes (Hendryx et al., 2020). Likewise, the Dutch HELIUS cohort study of a multi-ethnic population reported unhealthy lifestyle patterns were associated with higher risks of developing type 2 diabetes (van Etten et al., 2020). Despite the differences in risk factors and patterns considered that preclude direct comparisons between previous evidence and our results, taken together, these findings support an important role of lifestyle patterns in the development of type 2 diabetes.

The classic approach of studying single lifestyle factors usually assumes independent effects between each lifestyle factor, but does not account for their interrelations (Davis et al., 2019, Ding et al., 2015, Hendryx et al., 2020, Luo et al., 2021, Meader et al., 2016, Noble et al., 2015, van Etten et al., 2020). Although further investigation is warranted, we did observe that the risks related to different lifestyle patterns were neither additive nor proportionate to the number of risk factors present, especially compared with the effect sizes when studying each lifestyle factor separately (**Supplementary** Table S6). Notably, the “couch potato group” was not associated with risk of type 2 diabetes, especially after adjustment for BMI. This counterintuitive finding suggests that BMI may play an important role in the studied associations for participants from this lifestyle pattern group. As such, the average effects estimated for a single lifestyle risk factor may not be accurate for a substantial proportion of the study population. Alternatively, a lifestyle pattern may therefore be a proxy for an underlying behavioral variable that is not measured, but nevertheless relevant.

### 4.2 Methodological considerations

Our study was conducted in a single cohort, albeit large. Accordingly, the generalizability and reproducibility of the current lifestyle pattern analysis require further substantiation from independent cohorts. Various lifestyle patterns have been identified but in limited number of studies. At least partly, this is due to the heterogeneity of the source data, namely, numbers and categorization of lifestyle factors in different studies. Nevertheless, true differences in lifestyle patterns may exist between different populations. Analysis of differences and similarities in lifestyle patterns between populations would be highly relevant for identifying generic as well as specific patterns. So far, patterns primarily characterized by minimal risk behaviors, maximal risk behaviors, and poor diet combined with low physical activity were commonly identified. Patterns characterized by risk drinking generally showed large variations in its coexisting lifestyle risk factors across studies, which may be partly attributed to the lack of an evidence-based definition for that (Davis et al., 2019, Hendryx et al., 2020, Luo et al., 2021, Noble et al., 2015, van Etten et al., 2020, Watts et al., 2016). Using a normalized lifestyle evaluation scheme may therefore benefit the reproducibility and generalizability of the identified patterns to other populations.

### 4.3 Implications for public health prevention

In our analysis, participants from the “healthy lifestyle group” formed the largest group (44.3 %), although conspicuously their lifestyles were still not entirely optimal. Nevertheless, our analysis on lifestyle-related disease burden did show that substantial public health benefits could be obtained. For instance approximately one third of the diabetes cases in the “unhealthy lifestyle group” could be preventable, if participants in this group had the same lifestyle pattern as the “healthy lifestyle group” (**Supplementary** Table S5).

Current evidence supports the relevance of targeting multiple lifestyle risk factors simultaneously (Meader et al., 2016, Noble et al., 2015). Although certain efforts in diabetes prevention have been made on improving diet quality and physical activity, other lifestyle risk factors and within-population heterogeneity in the distribution of lifestyle factors have often been overlooked (Kivela et al., 2020). As observed in our population, lifestyle factors may coexist with each other in a counterintuitive manner. The “couch potato group”, characterized by excessive TV watching, also had the highest level of non-occupational MVPA. The differential risks found for each lifestyle pattern group also further emphasize the importance and relevance of considering different lifestyle patterns when designing lifestyle programs, rather than adopting the generic one-size-fits-all approach.

### 4.4 Strengths and limitations

Strengths of our study include a large sample size and the availability of data on TV watching time as an emerging lifestyle factor. Sensitivity analyses ensured the robustness of our findings. We exclusively studied lifestyle risk factors without conflation of lifestyle with its health outcomes (e.g., obesity status). However, a number of limitations are worth mentioning. First, over-reporting of healthy lifestyle behaviors due to social-desirability is possible (Newell et al., 1999). Nevertheless, in our study this over-reporting might mainly compromise the discrimination power of the identification of lifestyle clusters. Second, possible changes in lifestyle behaviors might be relevant but were not assessed. Third, as the Lifelines cohort mainly consists of participants in the northern Netherlands, it might not be possible to extrapolate our results to other population groups. Furthermore, in LCA analysis, the assignment of lifestyle pattern group for individuals was based on their highest posterior probability class membership, which unfortunately cannot account for the uncertainty of the classification. Finally, we could not analyze the potential impacts of lost to follow-up (23.0 %) among eligible participants. Nonetheless, the baseline characteristics of those who had no follow-up data were comparable with the study population, except for some minor differences (**Supplementary** Table S7). Simulation studies suggested that such attrition bias may only have limited influences on estimates of associations in cohort studies (Howe et al., 2013, Peters et al., 2012).

## 5 Conclusions

In conclusion, focusing on five lifestyle factors, namely smoking, overall diet quality, TV watching time, physical activity, and risk drinking, we identified five groups of individuals with different lifestyle patterns using a data-driven approach in a large population-based sample. These five lifestyle patterns were differentially associated with risk of developing type 2 diabetes. The clustering of lifestyle risk factors extends previous knowledge that those lifestyle factors tend to cluster, particularly in behavioral patterns within a general and heterogeneous population. Our findings pave the way for a more effective strategy for public health prevention for type 2 diabetes through targeting multiple lifestyle risk factors simultaneously.

## 6 Ethics approval

The Lifelines cohort study is conducted according to the principles of the Declaration of Helsinki and in accordance with the research code of the University Medical Center Groningen (approval number 2007/152). All participants received detailed information about the Lifelines cohort study and signed informed consent.

## Data availability

The manuscript is based on the data from the Lifelines cohort study. Lifelines adheres to standards for data availability. The data catalogue of the Lifelines cohort study is publicly accessible at https://www.lifelines.nl. All international researchers can obtain data at the Lifelines research office (research@lifelines.nl), for which a fee is required. The Lifelines research system allows access for reproducibility of the study results.

## Funding

This project has received funding from the European Union’s Horizon 2020 research and innovation program under the Marie Skłodowska-Curie grant agreement no. 754425. The Lifelines Biobank initiative has been made possible by subsidies from the Dutch Ministry of Health, Welfare and Sport, the Dutch Ministry of Economic Affairs, the University Medical Center Groningen (UMCG), University of Groningen, and the Provinces in the north of The Netherlands (Drenthe, Friesland, and Groningen). The funders had no role in study design, data collection and analysis, decision to publish, or preparation of the manuscript.

## CRediT authorship contribution statement

**Ming-Jie Duan:** Conceptualization, Methodology, Formal analysis, Data curation, Writing – original draft, Visualization, Project administration. **Louise H. Dekker:** Conceptualization, Writing – review & editing, Supervision, Project administration. **Juan-Jesus Carrero:** Writing – review & editing. **Gerjan Navis:** Conceptualization, Writing – review & editing, Supervision, Project administration, Funding acquisition.

## Declaration of Competing Interest

The authors declare that they have no known competing financial interests or personal relationships that could have appeared to influence the work reported in this paper.

## Data Availability

The authors do not have permission to share data.

## References

[b0005] Aune D., Norat T., Leitzmann M., Tonstad S., Vatten L.J. (2015). Physical activity and the risk of type 2 diabetes: a systematic review and dose-response meta-analysis. Eur. J. Epidemiol..

[b0010] Davis J.S., Banfield E., Lee H.Y., Peng H.L., Chang S., Wood A.C. (2019). Lifestyle behavior patterns and mortality among adults in the NHANES 1988–1994 population: a latent profile analysis. Prev. Med..

[b0015] de Vries H., van 't Riet J., Spigt M., Metsemakers J., van den Akker M., Vermunt J.K., Kremers S. (2008). Clusters of lifestyle behaviors: results from the Dutch SMILE study. Prev. Med..

[b0020] Ding C., O’Neill D., Bell S., Stamatakis E., Britton A. (2021). Association of alcohol consumption with morbidity and mortality in patients with cardiovascular disease: original data and meta-analysis of 48,423 men and women. BMC Medicine.

[b0025] Ding D., Rogers K., van der Ploeg H., Stamatakis E., Bauman A.E. (2015). Traditional and emerging lifestyle risk behaviors and all-cause mortality in middle-aged and older adults: evidence from a large population-based Australian cohort. PLoS Med..

[b0030] Duan M.J., Dekker L.H., Carrero J.J., Navis G. (2021). Blood lipids-related dietary patterns derived from reduced rank regression are associated with incident type 2 diabetes. Clin. Nutr..

[b0035] Duan M.J., Dekker L.H., Carrero J.J., Navis G. (2022). Using Structural Equation Modeling to Untangle Pathways of Risk Factors Associated with Incident Type 2 Diabetes: the Lifelines Cohort Study. Prev. Sci..

[b0040] Farhadnejad H., Teymoori F., Asghari G., Mokhtari E., Mirmiran P., Azizi F. (2022). The higher adherence to a healthy lifestyle score is associated with a decreased risk of type 2 diabetes in Iranian adults. BMC Endocr Disord.

[b0045] Hagenaars J.A., McCutcheon A.L. (2002).

[b0050] Halvorsen R.E., Elvestad M., Molin M., Aune D. (2021). Fruit and vegetable consumption and the risk of type 2 diabetes: a systematic review and dose–response meta-analysis of prospective studies. BMJ Nutr. Prev. Health.

[b0055] Hendryx M., Dinh P., Chow A., Kroenke C.H., Hingle M., Shadyab A.H., Garcia L., Howard B.V., Luo J. (2020). Lifestyle and psychosocial patterns and diabetes incidence among women with and without obesity: a prospective latent class analysis. Prev. Sci..

[b0060] Hofstetter H., Dusseldorp E., van Empelen P., Paulussen T.W.G.M. (2014). A primer on the use of cluster analysis or factor analysis to assess co-occurrence of risk behaviors. Prev. Med..

[b0065] Howe L.D., Tilling K., Galobardes B., Lawlor D.A. (2013). Loss to follow-up in cohort studies: bias in estimates of socioeconomic inequalities. Epidemiology.

[b0070] Kivela J., Wikstrom K., Virtanen E., Georgoulis M., Cardon G., Civeira F., Iotova V., Karuranga E., Ko W. (2020). Obtaining evidence base for the development of Feel4Diabetes intervention to prevent type 2 diabetes - a narrative literature review. BMC Endocr. Disord..

[b0075] Klijs B., Scholtens S., Mandemakers J.J., Snieder H., Stolk R.P., Smidt N. (2015). Representativeness of the LifeLines Cohort Study. PLoS ONE.

[b0080] Knott C., Bell S., Britton A. (2015). Alcohol consumption and the risk of type 2 diabetes: a systematic review and dose-response meta-analysis of more than 1.9 million individuals from 38 observational studies. Diabetes Care.

[b0085] Lanza S.T., Rhoades B.L. (2013). Latent class analysis: an alternative perspective on subgroup analysis in prevention and treatment. Prev. Sci..

[b0090] Luo J., Dinh P., Hendryx M., Li W., Robinson J., Margolis K.L. (2021). Risk Patterns and Mortality in Postmenopausal Women Using Latent Class Analysis. Am. J. Prev. Med..

[b0095] Maghsoudi Z., Ghiasvand R., Salehi-Abargouei A. (2016). Empirically derived dietary patterns and incident type 2 diabetes mellitus: a systematic review and meta-analysis on prospective observational studies. Public Health Nutr..

[b0100] Meader N., King K., Moe-Byrne T., Wright K., Graham H., Petticrew M., Power C., White M., Sowden A.J. (2016). A systematic review on the clustering and co-occurrence of multiple risk behaviours. BMC Public Health.

[b0105] Molag M.L., de Vries J.H., Duif N., Ocke M.C., Dagnelie P.C., Goldbohm R.A., van't Veer P. (2010). Selecting informative food items for compiling food-frequency questionnaires: comparison of procedures. Br. J. Nutr..

[b0110] Morris L.J., D'Este C., Sargent-Cox K., Anstey K.J. (2016). Concurrent lifestyle risk factors: clusters and determinants in an Australian sample. Prev. Med..

[b0115] Newell S.A., Girgis A., Sanson-Fisher R.W., Savolainen N.J. (1999). The accuracy of self-reported health behaviors and risk factors relating to cancer and cardiovascular disease in the general population: a critical review. Am. J. Prev. Med..

[b0120] Newson R.B. (2013). Attributable and Unattributable Risks and Fractions and other Scenario Comparisons. The STATA Journal.

[b0125] Noble N., Paul C., Turon H., Oldmeadow C. (2015). Which modifiable health risk behaviours are related? A systematic review of the clustering of Smoking, Nutrition, Alcohol and Physical activity ('SNAP') health risk factors. Prev. Med..

[b0130] Nylund K.L., Asparouhov T., Muthén B.O. (2007). Deciding on the Number of Classes in Latent Class Analysis and Growth Mixture Modeling: A Monte Carlo Simulation Study. Structural Equation Modeling: A Multidisciplinary Journal.

[b0135] Pan A., Wang Y., Talaei M., Hu F.B., Wu T. (2015). Relation of active, passive, and quitting smoking with incident type 2 diabetes: a systematic review and meta-analysis. Lancet Diabetes Endocrinol..

[b0140] Patterson R., McNamara E., Tainio M., de Sá T.H., Smith A.D., Sharp S.J., Edwards P., Woodcock J., Brage S. (2018). Sedentary behaviour and risk of all-cause, cardiovascular and cancer mortality, and incident type 2 diabetes: a systematic review and dose response meta-analysis. Eur. J. Epidemiol..

[b0145] Peters S.A., Bots M.L., den Ruijter H.M., Palmer M.K., Grobbee D.E., Crouse J.R., O'Leary D.H., Evans G.W., Raichlen J.S. (2012). Multiple imputation of missing repeated outcome measurements did not add to linear mixed-effects models. J. Clin. Epidemiol..

[b0150] Poortinga W. (2007). The prevalence and clustering of four major lifestyle risk factors in an English adult population. Prev. Med..

[b0155] Rabe-Hesketh S., Skrondal A. (2008). Classical latent variable models for medical research. Stat. Methods Med. Res..

[b0160] Scholtens S., Smidt N., Swertz M.A., Bakker S.J., Dotinga A., Vonk J.M., van Dijk F., van Zon S.K., Wijmenga C. (2015). Cohort Profile: LifeLines, a three-generation cohort study and biobank. Int. J. Epidemiol..

[b0165] Siebelink E., Geelen A., de Vries J.H. (2011). Self-reported energy intake by FFQ compared with actual energy intake to maintain body weight in 516 adults. Br. J. Nutr..

[b0170] Streppel M.T., de Vries J.H., Meijboom S., Beekman M., de Craen A.J., Slagboom P.E., Feskens E.J. (2013). Relative validity of the food frequency questionnaire used to assess dietary intake in the Leiden Longevity Study. Nutr. J..

[b0175] Sun H., Saeedi P., Karuranga S., Pinkepank M., Ogurtsova K., Duncan B.B., Stein C., Basit A., Chan J.C.N. (2022). IDF Diabetes Atlas: Global, regional and country-level diabetes prevalence estimates for 2021 and projections for 2045. Diabetes Res. Clin. Pract..

[b0180] Tuomilehto J., Schwarz P., Lindstrom J. (2011). Long-term benefits from lifestyle interventions for type 2 diabetes prevention: time to expand the efforts. Diabetes Care.

[b0185] van Etten S., Crielaard L., Muilwijk M., van Valkengoed I., Snijder M.B., Stronks K., Nicolaou M. (2020). Lifestyle clusters related to type 2 diabetes and diabetes risk in a multi-ethnic population: The HELIUS study. Prev. Med..

[b0190] Vermunt, J.K., Magidson, J., 2005. LATENT GOLD 4.0 User's Guide. Statistical Innovations Inc., Belmont, Massachusetts, USA.

[b0195] Vinke P.C., Corpeleijn E., Dekker L.H., Jacobs D.R., Navis G., Kromhout D. (2018). Development of the food-based Lifelines Diet Score (LLDS) and its application in 129,369 Lifelines participants. Eur. J. Clin. Nutr..

[b0200] Watts P., Buck D., Netuveli G., Renton A. (2016). Clustering of lifestyle risk behaviours among residents of forty deprived neighbourhoods in London: lessons for targeting public health interventions. J Public Health (Oxf).

[b0205] Weggemans R.M., Backx F.J.G., Borghouts L., Chinapaw M., Hopman M.T.E., Koster A., Kremers S., van Loon L.J.C., May A. (2018). The 2017 Dutch Physical Activity Guidelines. Int J Behav Nutr Phys Act.

[b0210] Wendel-Vos G.C., Schuit A.J., Saris W.H., Kromhout D. (2003). Reproducibility and relative validity of the short questionnaire to assess health-enhancing physical activity. J. Clin. Epidemiol..

[b0215] World Health Organization (WHO), International Diabetes Federation (IDF) (2006).

[b0220] Zhang Y., Pan X.-F., Chen J., Xia L., Cao A., Zhang Y., Wang J., Li H., Yang K. (2020). Combined lifestyle factors and risk of incident type 2 diabetes and prognosis among individuals with type 2 diabetes: a systematic review and meta-analysis of prospective cohort studies. Diabetologia.

