# Supplementary material for: Lifestyle patterns and incident type 2 diabetes in the Dutch lifelines cohort study
Source: Prev Med Rep. 2022 Oct 3;30:102012. doi: 10.1016/j.pmedr.2022.102012 (PMC9551208; doi:10.1016/j.pmedr.2022.102012)
Supplement: Supplementary data 1 [file mmc1.docx]

**Lifestyle Patterns and Incident Type 2 Diabetes in the Dutch Lifelines Cohort Study**

**Supplementary Materials**

Ming-Jie Duan^1^, Louise H. Dekker^1,2^, Juan-Jesus Carrero^3^, Gerjan Navis^1^

^1^Department of Internal Medicine, University Medical Center Groningen, Groningen, The Netherlands;

^2^National Institute for Public Health and the Environment, Bilthoven, The Netherlands;

^3^Department of Medical Epidemiology and Biostatistics, Karolinska Institutet, Stockholm, Sweden.

Corresponding author: Ming-Jie Duan, m.duan@umcg.nl

**Supplementary Fig. S1 - Study flow chart**


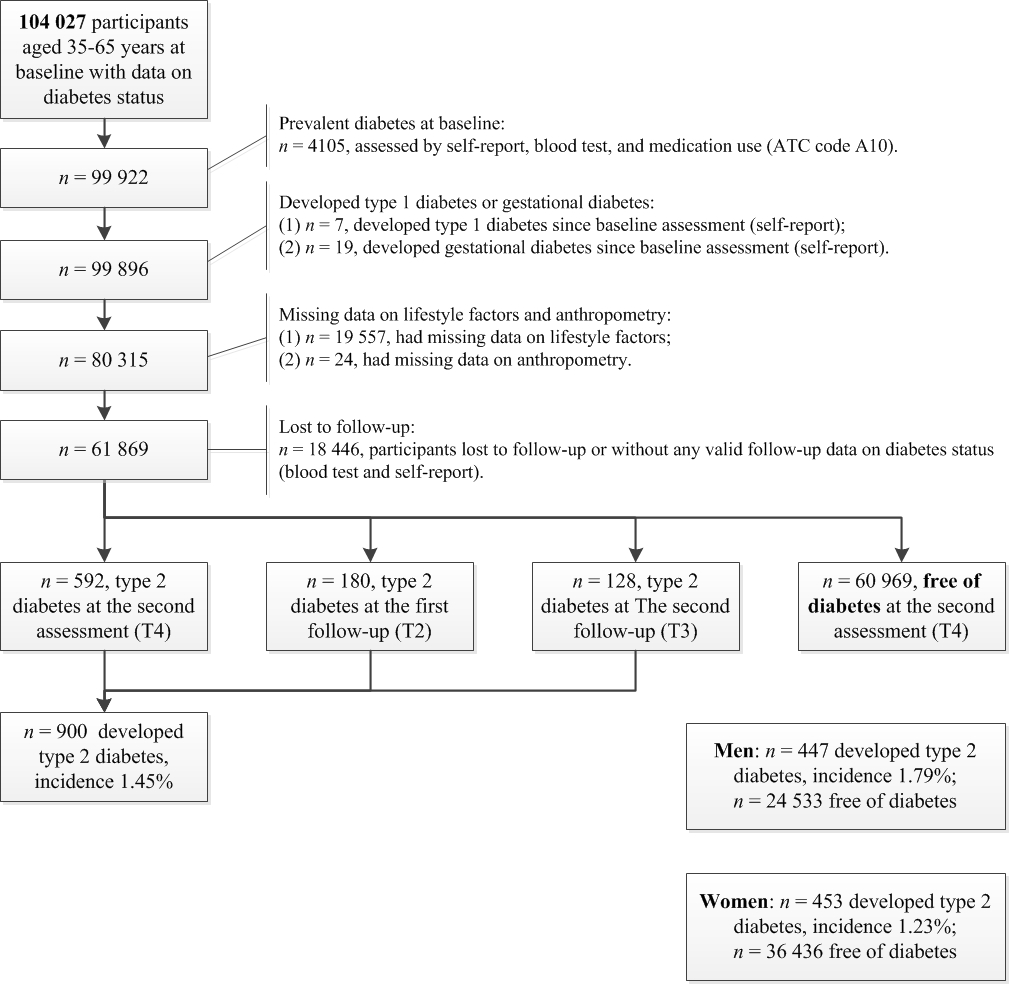


**Supplementary Table S1 - Model goodness-of-fit indices for latent class analysis with 3 through 9 classes tested**

|  | ***N*_par_** | **BIC (LL)^*^** | **AIC (LL)^*^** | **CAIC (LL)^*^** | ***L*^2^** | ***df*** | ***p*-value** | **LL** | **Class. Err^†^** | **Entropy (*R*^2^)^‡^** |
| --- | --- | --- | --- | --- | --- | --- | --- | --- | --- | --- |
| 3-cluster | 21 | 479000 | 478810 | 479021 | 568 | 50 | 4.9×10^-89^ | -239384 | 0.2681 | 0.4113 |
| 4-cluster | 28 | 478730 | 478477 | 478758 | 221 | 43 | 7.6×10^-26^ | -239211 | 0.2910 | 0.4825 |
| **5-cluster** | **35** | **478670** | **478353** | **478705** | **84** | **36** | **1.2×10^-5^** | **-239142** | **0.3089** | **0.4934** |
| 6-cluster | 42 | 478717 | 478338 | 478759 | 54 | 29 | 3.5×10^-3^ | -239127 | 0.3702 | 0.4222 |
| 7-cluster | 49 | 478764 | 478322 | 478813 | 24 | 22 | 0.36 | -239112 | 0.4220 | 0.3867 |
| 8-cluster | 56 | 478830 | 478324 | 478886 | 12 | 15 | 0.67 | -239106 | 0.4410 | 0.4067 |
| 9-cluster | 63 | 478908 | 478339 | 478971 | 13 | 8 | 0.11 | -239107 | 0.5060 | 0.3168 |

^*^BIC(LL), AIC(LL), and CAIC(LL) denotes Bayesian information criterion, Akaike information criterion, and consistent Akaike information criterion, respectively. LL indicates these indices are log likelihood statistics with the number of parameters adjusted. These three model goodness-of-fit indices weight fit and parsimony by adjusting the log likelihood to account for the number of parameters in the model. Generally, in model comparison, lower values are preferred as they indicate a better model fit. Specifically, CAIC-LL has an advantage over AIC-LL since CAIC-LL adds an additional penalty for larger numbers of classes, while AIC-LL frequently led to overfitting. Additionally, BIC-LL is considered superior to AIC-LL, as the former penalizes complexity of the latent model. In the current study, our best-fitting model selection was chiefly based on BIC-LL value, and also made reference to other model fit indices.

^†^Class. Err (classification error) estimates the proportion of cases that are likely to be misclassified into the wrong latent class group. In model comparison, lower values (closer to 0) are preferred as they indicate a low chance of class membership misclassification.

^‡^Entropy (*R*^2^) is a pseudo *R*^2^ statistic that indicates how well one can predict the class membership based on the observed variables, namely, this value assesses the accuracy that how distinct the classes derived are from one another. In model comparison, larger values (closer to 1) are preferred.

[1] Hagenaars, J. A., & McCutcheon, A. L. (Eds.). (2002). Applied latent class analysis. Cambridge University Press.

[2] Vermunt, J. K., & Magidson, J. (2004). Latent class analysis. The sage encyclopedia of social sciences research methods, 2, 549-553.

[3] Nylund, Karen L., Tihomir Asparouhov, and Bengt O. Muthén. Deciding on the number of classes in latent class analysis and growth mixture modeling: A Monte Carlo simulation study. Structural Equation Modeling 14.4 (2007): 535-569.

**Supplementary Table S2 - Estimated probabilities of adhering to examined lifestyle factors for each identified lifestyle pattern group using latent class analysis**

|  | **Healthy lifestyle group** | **Poor diet and low physical activity group** | **Unhealthy lifestyle group** | **Couch potato group** | **Risk drinker group** |
| --- | --- | --- | --- | --- | --- |
| Cluster size (*n*) | 27,413 | 13,846 | 12,031 | 4726 | 3853 |
| Cluster size (%) | 44.3 | 22.4 | 19.5 | 7.6 | 6.2 |
| Risk drinking | 0.0011 | 0.0958 | 0.2866 | 0.2526 | 0.9591 |
| Never smoker | 0.5242 | 0.8256 | 0.0069 | 0.2343 | 0.2443 |
| Former smoker | 0.3984 | 0.1736 | 0.3438 | 0.6805 | 0.5937 |
| Current smoker | 0.0774 | 0.0007 | 0.6493 | 0.0851 | 0.1620 |
| Lifelines diet score tertiles | | | | | |
| First | 0.1062 | 0.5005 | 0.5098 | 0.1791 | 0.1476 |
| Second | 0.3655 | 0.3790 | 0.3746 | 0.4157 | 0.3991 |
| Third | 0.5283 | 0.1205 | 0.1156 | 0.4052 | 0.4533 |
| Insufficient physical activity | 0.3023 | 0.5071 | 0.6272 | 0.2726 | 0.2649 |
| Excessive TV watching | 0.2356 | 0.4034 | 0.5286 | 0.9761 | 0.1267 |

**Supplementary Table S3 - Associations between lifestyle pattern groups and incident type 2 diabetes using the “unhealthy lifestyle group” as reference^*^**

|  | **Healthy lifestyle group** | **Poor diet and low physical activity group** | **Unhealthy lifestyle group** | **Couch potato group** | **Risk drinker group** |
| --- | --- | --- | --- | --- | --- |
| Cases/Population | 321 / 27,413 | 187 / 13,846 | 255 / 12,031 | 81 / 4726 | 56 / 3853 |
| Incidence, % | 1.17 | 1.35 | 2.12 | 1.71 | 1.45 |
| Incidence rates, per 1000 person-years | 3.51 | 4.09 | 6.42 | 5.17 | 4.25 |
| Model 1 | 0.50 (0.43, 0.59) | 0.74 (0.61, 0.89) | 1.00 (ref) | 0.64 (0.50, 0.83) | 0.50 (0.37, 0.67) |
| Model 2 | 0.56 (0.47, 0.66) | 0.78 (0.64, 0.94) |  | 0.65 (0.51, 0.85) | 0.58 (0.43, 0.78) |
| Model 3 | 0.61 (0.52, 0.72) | 0.74 (0.61, 0.90) |  | 0.65 (0.50, 0.84) | 0.67 (0.49, 0.90) |
| Model 4 | 0.61 (0.52, 0.73) | 0.74 (0.61, 0.90) |  | 0.65 (0.50, 0.84) | 0.68 (0.50, 0.92) |
| Model 5 | 0.66 (0.54, 0.81) | 0.84 (0.67, 1.04) |  | 0.64 (0.50, 0.83) | 0.68 (0.50, 0.93) |

^*^All models: HRs (95% CI) derived from multivariate Cox proportional hazards models. Model 1 was adjusted for age, sex and total energy intake, *n* = 61,869; model 2 was adjusted for model 1 covariates plus education, *n* = 61,714; model 3 was adjusted for model 2 covariates plus BMI, *n* = 61,714; model 4 was adjusted for model 3 covariates plus family history of diabetes, *n* = 61,714; model 5 was adjusted for model 4 covariates plus blood glucose level at baseline, *n* = 61,512.

**Supplementary Table S4 - Associations between lifestyle pattern groups and incident type 2 diabetes excluding participants who had less than 12-month follow-up**

|  | **Multivariate model^*^** |
| --- | --- |
| Healthy lifestyle group | 1.00 (ref) |
| Poor diet and low physical activity group | 1.26 (1.02, 1.54) |
| Unhealthy lifestyle group | 1.51 (1.23, 1.85) |
| Couch potato group | 0.99 (0.77, 1.27) |
| Risk drinker group | 1.04 (0.77, 1.40) |

^*^Multivariate model: HRs (95% CI) derived from multivariate Cox proportional hazards model, adjusted for age, sex, total energy intake, education, BMI, family history of diabetes, and blood glucose level at baseline, *n* = 61,028.

**Supplementary Table S5 - Fraction of type 2 diabetes cases preventable (population attributable fraction, %) for each lifestyle pattern group, using the “healthy lifestyle group” as reference^*^**

|  | **Basic model** | **Multivariate model 1** | **Multivariate model 2** |
| --- | --- | --- | --- |
| Healthy lifestyle group | Reference | | |
| Poor diet and low physical activity group | 29.2 (15.1, 41.0) | 14.7 (-2.4, 28.9) | 12.8 (-4.1, 26.9) |
| Unhealthy lifestyle group | 48.6 (39.4, 56.3) | 35.6 (23.8, 45.6) | 22.7 (9.2, 34.2) |
| Couch potato group | 20.3 (-1.8, 37.5) | 1.2 (-26.4, 22.7) | -16.8 (-47.1, 7.3) |
| Risk drinker group | 6.8 (-23.7, 29.8) | 15.1 (-12.7, 36.0) | -5.6 (-38.3, 19.3) |
| Overall | 22.1 (15.0, 28.6) | 14.1 (6.2, 21.4) | 7.2 (-1.1, 14.8) |

^*^All models were estimated with multivariate logistic regression models. Basic model was adjusted for age, sex and total energy intake, *n* = 61,869; multivariate model 1 was adjusted for basic model covariates plus education, BMI, and family history of diabetes, *n* = 61,714; multivariate model 2 was adjusted for multivariate model 1 covariates plus blood glucose level at baseline, *n* = 61,512. Population attributable fractions were derived based on the ORs estimated from the aforementioned logistic regression models.

**Supplementary Table S6 - Associations between single lifestyle risk factors and incident type 2 diabetes^*^**

|  | **Singly adjusted** | **Mutually adjusted 1** | **Mutually adjusted 2** |
| --- | --- | --- | --- |
| Insufficient physical activity | 1.19 (1.04, 1.36) | 1.13 (0.98, 1.29) | 1.13 (0.97, 1.31) |
| Lifelines diet score |  |  |  |
| Middle tertile | 1.10 (0.93, 1.30) | 1.06 (0.90, 1.25) | 0.99 (0.83, 1.19) |
| Lowest tertile | 1.44 (1.21, 1.72) | 1.34 (1.12, 1.60) | 1.20 (0.99, 1.45) |
| Excessive TV watching | 1.41 (1.22, 1.62) | 1.36 (1.18, 1.56) | 1.37 (1.17, 1.60) |
| Smoking |  |  |  |
| Former smoker | 1.15 (0.99, 1.34) | 1.14 (0.98, 1.33) | 0.99 (0.85, 1.16) |
| Current smoker | 1.57 (1.31, 1.88) | 1.45 (1.20, 1.74) | 1.21 (0.96, 1.53) |
| Risk drinker | 1.16 (0.98, 1.37) | 1.06 (0.89, 1.26) | 1.03 (0.87, 1.23) |

^*^All models: HRs (95% CI) derived from multivariate Cox proportional hazards model, adjusted for age, sex, total energy intake, education, BMI, and family history of diabetes, *n* = 61,714. Singly adjusted: models were adjusted for each lifestyle factor separately. Mutually adjusted 1: models were adjusted all lifestyle factors simultaneously. Mutually adjusted 2: models adjusted for all abovementioned covariates plus blood glucose level at baseline, *n* = 61,512. Reference for each lifestyle risk factor for the models estimated: sufficient physical activity (≥150 min/week non-occupational moderate-to-vigorous physical activity); highest tertile of the Lifelines diet score; non-excessive TV watching (first and second tertile of TV watching time); never smoker; and non-risk drinker (≤15g alcohol/day).

**Supplementary Table S7 - Comparisons of baseline characteristics between included participants and those who had no follow-up data**

|  | **Lost to follow-up** | **Study sample** |
| --- | --- | --- |
| Number of participants | 18 446 | 61 869 |
| Age, yrs | 46.5±7.5 | 48.2±7.9 |
| Sex - women, % | 58.6 | 59.6 |
| Education, % |  |  |
| Low | 33.6 | 28.8 |
| Middle | 39.1 | 39.6 |
| High | 27.0 | 31.3 |
| Fasting glucose, mmol/L | 4.95±0.51 | 4.95±0.50 |
| HbA_1c_, % | 5.52±0.30 | 5.54±0.30 |
| Systolic blood pressure, mmHg | 124.8±15.0 | 125.5±15.0 |
| Diastolic blood pressure, mmHg | 74.6±9.6 | 74.8±9.4 |
| BMI, kg/m^2^ | 26.4±4.4 | 26.1±4.1 |
| WHR | 0.91±0.08 | 0.91±0.08 |
| Family history of diabetes, % | 12.4 | 8.8 |
| Total energy intake, kcal/day | 2061±626 | 2062±590 |
| Lifeline diet score | 23.3±6.0 | 24.0±5.9 |
| Risk drinking, % | 16.6 | 16.5 |
| Alcohol intake, g/day | 3.8 (0.7, 10.7) | 4.5 (0.9, 11.0) |
| Meeting physical recommendation (150 min/week MVPA) | 54.8 | 59.2 |
| MVPA, min/week | 180 (60, 360) | 180 (60, 360) |
| TV watching time, hrs/day | 2.5±1.4 | 2.4±1.3 |
| Smoking, % |  |  |
| Never | 42.6 | 44.9 |
| Former | 32.2 | 37.4 |
| Current | 25.2 | 17.7 |
